# Adjunctive nano‐curcumin therapy improves inflammatory and clinical indices in children with cystic fibrosis: A randomized clinical trial

**DOI:** 10.1002/fsn3.3323

**Published:** 2023-03-28

**Authors:** Saeedeh Talebi, Andrew S. Day, Mahammad Safarian, Seyed Javad Sayedi, Mahmood Reza Jaafari, Zahra Abbasi, Hanieh Barghchi, Hamid Reza Kianifar

**Affiliations:** ^1^ Department of Nutrition, Faculty of Medicine Mashhad University of Medical Sciences Mashhad Iran; ^2^ Department of Pediatrics University of Otago Christchurch New Zealand; ^3^ Department of Pediatrics Mashhad University of Medical Sciences Mashhad Iran; ^4^ Nanotechnology Research Center, Pharmaceutical Technology Institute Mashhad University of Medical Sciences Mashhad Iran; ^5^ Akbar Clinical Research and Development Unit Mashhad University of Medical Sciences Mashhad Iran

**Keywords:** child, curcumin, cystic fibrosis, inflammation, treatment outcome

## Abstract

Inflammation may develop due to internal dysfunction of the cystic fibrosis transmembrane conductance regulator (CFTR) protein or external factors in patients with cystic fibrosis (CF). This prospective randomized clinical trial aimed to ascertain the effects of nano‐curcumin as an anti‐inflammatory agent and a CFTR modulator on clinical and inflammatory markers in children with CF. Children with CF were randomly assigned to receive daily curcumin or a placebo for 3 months. The primary outcome measure was to evaluate inflammatory indices, nasopharyngeal swab analysis, and clinical assessments via spirometry, anthropometric measurements, and quality of life (QOL) analysis. Sixty children were included. Intra‐group changes comparison showed that curcumin decreased the level of high‐sensitivity C‐reactive protein (hs‐CRP) (median: −0.31 mg/L, IQR: −1.53 to 0.81; *p* = .01) and fecal calprotectin level (−29 μg/g, −57.5 to 11.5; *p* = .03), also increased the level of interleukin (IL)‐10 (6.1 pg/mL, 4.5–9; *p* = .01). Moreover, curcumin improved the overall QOL and the subscales of the questionnaire. Inter‐group changes comparison depicted the number of *Pseudomonas* colonies reduced by about 52% in the curcumin group and gained weight by about 16% (*p* > .05). Nano‐curcumin seems to be considered as an effective nutritional supplement on hs‐CRP, IL‐10, fecal calprotectin levels, and improving QOL in patients with CF.

## Graphical abstract

This trial aimed to ascertain the effects of nano‐curcumin as an anti‐inflammatory agent and a cystic fibrosis transmembrane conductance regulator modulator on clinical and inflammatory markers in children with cystic fibrosis (CF). Nano‐curcumin seems to be considered as an effective nutritional supplement on hs‐CRP, IL‐10, fecal calprotectin level and improving quality of life on patients with CF.

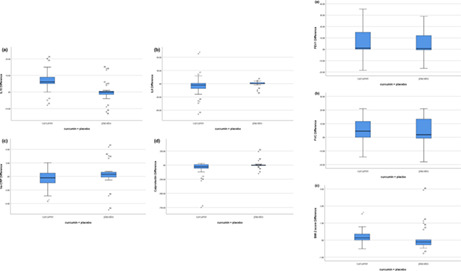

## 1 INTRODUCTION

Cystic fibrosis (CF) is caused by a mutation in the CF transmembrane conductance regulator (CFTR) gene (Dorwart et al., 2004).

Persistent inflammation is primarily caused by protein deficiency, secondary to external factors, including persistent bacterial infections. Since inflammation has been proposed as an underlying cause of CF, major efforts have been made to propose efficient pharmacological and herbal anti‐inflammatory therapies (Cantin et al., 2015; Mitri et al., 2020; Taylor‐Cousar et al., 2010). Curcumin, which is the most active ingredient in the turmeric root, has a significant effect on reducing inflammation (Dey et al., 2016; Lelli et al., 2017). Besides, it can be effective in the correction of deltaF508 mutations, associated with misfolding of the CFTR protein and improved protein function (Lipecka et al., 2006).

Curcumin acts as an allosteric agent in improving the function of the CFTR protein among the W1282X mutation (Wang et al., 2016). Moreover, the potential effect of curcumin on the CFTR protein has been shown in patients with aG551D mutation (Yu et al., 2011). In addition to the primary effects of curcumin on the CFTR protein, it has been reported to be effective as an antimicrobial agent, enhancing the effects of routine antimicrobial therapies; it can be even used alone, especially for reducing *Pseudomonas aeruginosa* biofilms (Karaman et al., 2013).

In addition to CF, the effects of curcumin on various adult diseases have been evaluated and confirmed with no serious side effects (Ahmadi et al., 2021; Heshmati et al., 2021; Rahimi et al., 2016), but studies on children was limited.

Because curcumin is a poorly water‐soluble substance with a short half‐life and low bioavailability, different compounds have been proposed to improve its bioavailability by increasing the rate of absorption from the gastrointestinal tract and reducing the hepatic metabolism of this substance (Cartiera et al., 2010; Flora et al., 2013; Yallapu et al., 2015). A nano‐curcumin micelle can be effective in improving its effects. This study was conducted to answer the question of whether or not these effects are also visible in the clinical setting.

## 2 METHODS

### 2.1 Study design

This study was designed as a prospective, double‐blind, single‐center, parallel‐group, randomized, placebo‐controlled clinical trial. The trial was registered at the Iranian Registry of Clinical Trials (Number: 20200705048018N1). The full study protocol is available online (Talebi, Safarian, et al., 2021) https://trialsjournal.biomedcentral.com/articles/10.1186/s13063‐021‐05224‐6.

### 2.2 Study population

The study population consisted of children with known CF seen at the Cystic Fibrosis Clinic of Akbar Children's Hospital in Mashhad, Iran. Children were screened for eligibility and recruited at the clinic. The inclusion criteria were as follows: (1) one or more typical phenotypic features of CF (e.g. chronic pulmonary disease, chronic sinusitis, characteristic gastrointestinal and nutritional abnormalities, salt loss syndrome, or obstructive azospermia), in addition to elevated sweat chloride concentrations at least on two or more occasions or two mutations known to cause CF on separate alleles; (2) age range of 5–18 years; (3) pulmonary or gastrointestinal involvement; (4) ability to perform spirometry maneuvers, with minimum forced expiratory volume in 1 second (FEV1) ≥30% compared to the normal population with the same age, gender, and height; (5) oxygen saturation ≥90% based on pulse oximetry at room temperature; and (6) informed consent for participation in the study.

Potential participants were excluded if they had (1) cardiovascular, hepatic, or renal failure; (2) celiac disease or rheumatoid arthritis; (3) acute pulmonary exacerbation requiring hospitalization in the past 4 weeks; or (4) a current acute respiratory tract infection. Informed consent was obtained from children and their parents at the time of enrollment.

### 2.3 Study intervention

Nano‐curcumin (Exir Nano Sina Co.) was prepared as a nano‐micelle (70 mg drops in 1 mL), and the placebo was prepared with the same color, taste, and odor. The control group received the placebo (similar components of nano‐micelle curcumin drop without curcumin) (Hatamipour et al., 2019).

The highest acceptable dose of nano‐curcumin in adults was previously shown to be 80 mg daily (Jazayeri‐Tehrani et al., 2019). Accordingly, the dosage in the current study was adjusted for body surface area, using the following equation:

$$x=\frac{80\;\mathrm{mg}\times \text{body surface area of each children}\;}{1.73}$$

The curcumin and placebo containers were labeled A and B respectively by the manufacturer and made available to the subjects in a double‐blind manner. Curcumin or placebo was administered orally alone or with sweetened water (using sucrose) once daily at bedtime at a fixed dose for 3 months.

### 2.4 Randomization

A table of random numbers was used to allocate children to one of the two groups using stratified randomization, based on disease severity via spirometry at a 40% FEV1 cutoff value. Sealed envelopes were used for allocation concealment. Further randomization details were included in the published study protocol (Talebi, Safarian, et al., 2021).

### 2.5 Outcomes

The primary outcomes of this trial were changes in systemic inflammatory markers (interleukin (IL)‐6, IL‐10 and high‐sensitivity C‐reactive protein [hs‐CRP]), changes in pulmonary inflammation (neutrophil count, bacterial culture, and viral culture of nasopharyngeal secretions) and changes in gastrointestinal inflammation (fecal calprotectin) over the duration of the study period. The secondary outcomes included changes in pulmonary function (spirometry), anthropometric indices, and quality of life (QOL) scores over the duration of the study. The validated Cystic Fibrosis Questionnaire (CFQ) was utilized to determine QOL scores (Talebi et al., 2022; Talebi, Sayedi, et al., 2021).

### 2.6 Statistical analysis

A sample size of 30 subjects in each group was determined by G‐power analyzer (Version, Company, City, Country), using hsCRP indices as an effect size and Deff (design effect) for stratified sample, at a power of 80%, a significance level of .05, and a drop‐out rate of 10%. Data analysis was performed in SPSS version 16 (Company, City, Country). Data are expressed as the frequency for qualitative variables, mean and standard deviation (SD) for normally distributed continuous variables, and median and interquartile range (IQR) for other variables. Intergroup differences were evaluated using the paired *t‐*test for normally distributed continuous variables and the Wilcoxon rank‐sum test for other variables. Intra‐group differences were also assessed using the independent *t*‐test for normally distributed continuous variables and the Mann–Whitney *U* test for other variables. Mean changes in the secondary outcomes were adjusted for the baseline data, using analysis of covariance (ANCOVA). In all statistical analyses, a *p*‐value <.05 was considered significant.

## 3 RESULTS

### 3.1 Study recruitment

**FIGURE 1:**
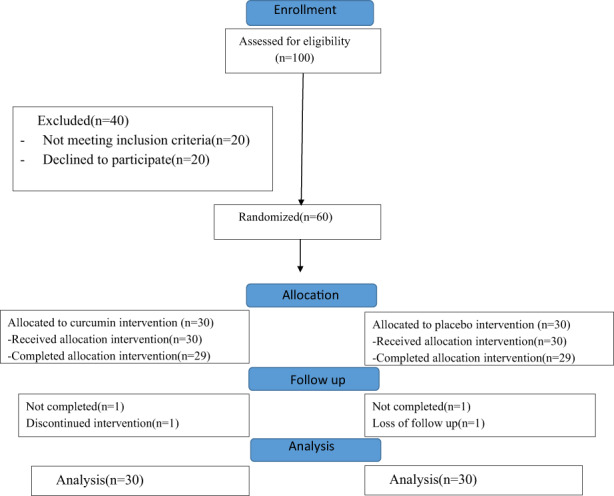
Screening, randomization, follow‐up, analysis.

Of the 100 patients screened, 60 patients met the eligibility criteria and were randomized into the intervention or placebo group (Figure 1). One subject in the placebo group died, and one subject in the curcumin group dropped out due to intolerance to curcumin due to its taste. The data of the 60 subjects were included in the final analysis by intention to treat analysis. The two groups had similar demographic characteristics (Table 1).

**TABLE 1:** Demographic characteristics of intervention (curcumin) and placebo group.

Variables	Subgroup	Mean (SD)	*p* Value^a^
Age (year)	Curcumin	5.06 ± 11.66	.32
	Placebo	6.3 ± 13.29	
Gender percent (number)			
Girl	Curcumin	51.7% (15)	.2
Boy		48.3% (14)	
Girl	Placebo	31.6% (6)	
Boy		68.4% (13)	
Weight(kg)	Curcumin	28.98 ± 12.78	.15
	Placebo	17.93 ± 36.02	
Height(cm)	Curcumin	18.69 ± 134.53	.12
	Placebo	22.06 ± 144.52	
BMI	Curcumin	2.58 ± 15.15	.89
	Placebo	4.35 ± 16.44	
BMI (*z* score)	Curcumin	1.63 ± −1.7	.49
	Placebo	1.57 ± −1.78	
MUAC (cm)	Curcumin	3.87 ± 18.97	.23
	Placebo	0.17 ± 0.17	

Abbreviations: BMI, body mass index; MUAC, mid‐upper arm circumference; SD, Standard deviation.

^a^ Independent *t*‐test.

### 3.2 Bioavailability

The maximum daily dose of curcumin in the current study was 80 mg. The encapsulation efficiency of curcuminoids in nano‐micelles is almost 100% and the mean diameter of nano‐micelles is around 10 nm, according to dynamic light scattering. The oral absorption of Sina Curcumin™ is at least 50 times higher than the absorption of the conventional powder of curcumin in mice (Hatamipour et al., 2019). Consequently, pharmacokinetic studies are needed to evaluate the plasma level of curcumin in this population.

### 3.3 Comparison of inflammatory status

**FIGURE 2:**
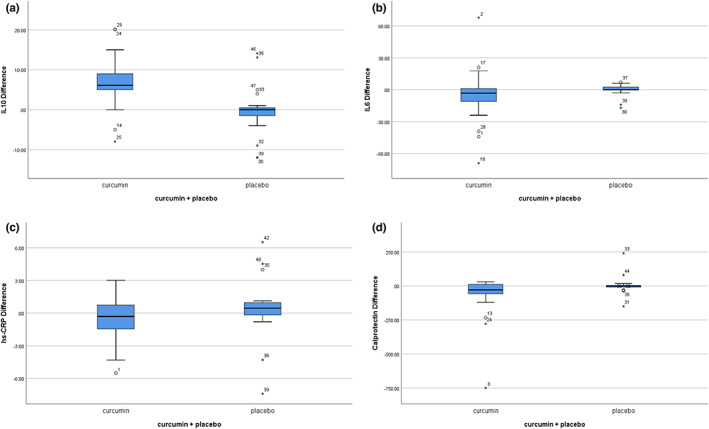
Evaluation of inflammatory status in the two groups before and after the intervention: Adjusted mean changes of plasma IL10 in the curcumin and placebo groups showed significant improvement in serum levels of IL‐10 in the curcumin group with the use of the Mann–Whitney *U* test (a), adjusted mean changes of plasma IL‐6 in curcumin and placebo groups with the use of a Mann–Whitney *U* test showed no significant differences in the curcumin and placebo groups (b), adjusted mean changes of plasma hs‐CRP in the curcumin and placebo groups showed that the intra‐group had significant improvement in the level of plasma hs‐CRP in the curcumin group (c), adjusted mean changes of fecal calprotectin in the curcumin and placebo groups showed that fecal calprotectin significantly decreased in the curcumin group (d).

Median (and IQR) serum IL‐6 levels decreased by 3.2 pg/L (−11.5 to 1.1) in the curcumin group. IL‐6 levels did not decrease in the placebo group. However, between and within‐group differences were not significant (Figure 2b). Serum IL‐10 levels increased by 6.1 pg/L (4.5–9) in the curcumin group (*p* = .001), but not in the placebo group. Again, there were no between‐group differences (Figure 2a).

While median plasma hs‐CRP levels decreased by 0.31 mg/L (−1.53 to 0.81) in the curcumin group, levels increased by 0.46 (−0.33 to 1) in the control group (*p* = .01; Figure 2c). Furthermore, fecal calprotectin levels decreased by 29 μg/g (−57.5 to 11.5) in the intervention group (*p* = .001), with no change in the control group. The difference within the two groups was almost significant (*p* = .08) (Figure 2d; Table S1).

### 3.4 Comparison of clinical outcomes

**FIGURE 3:**
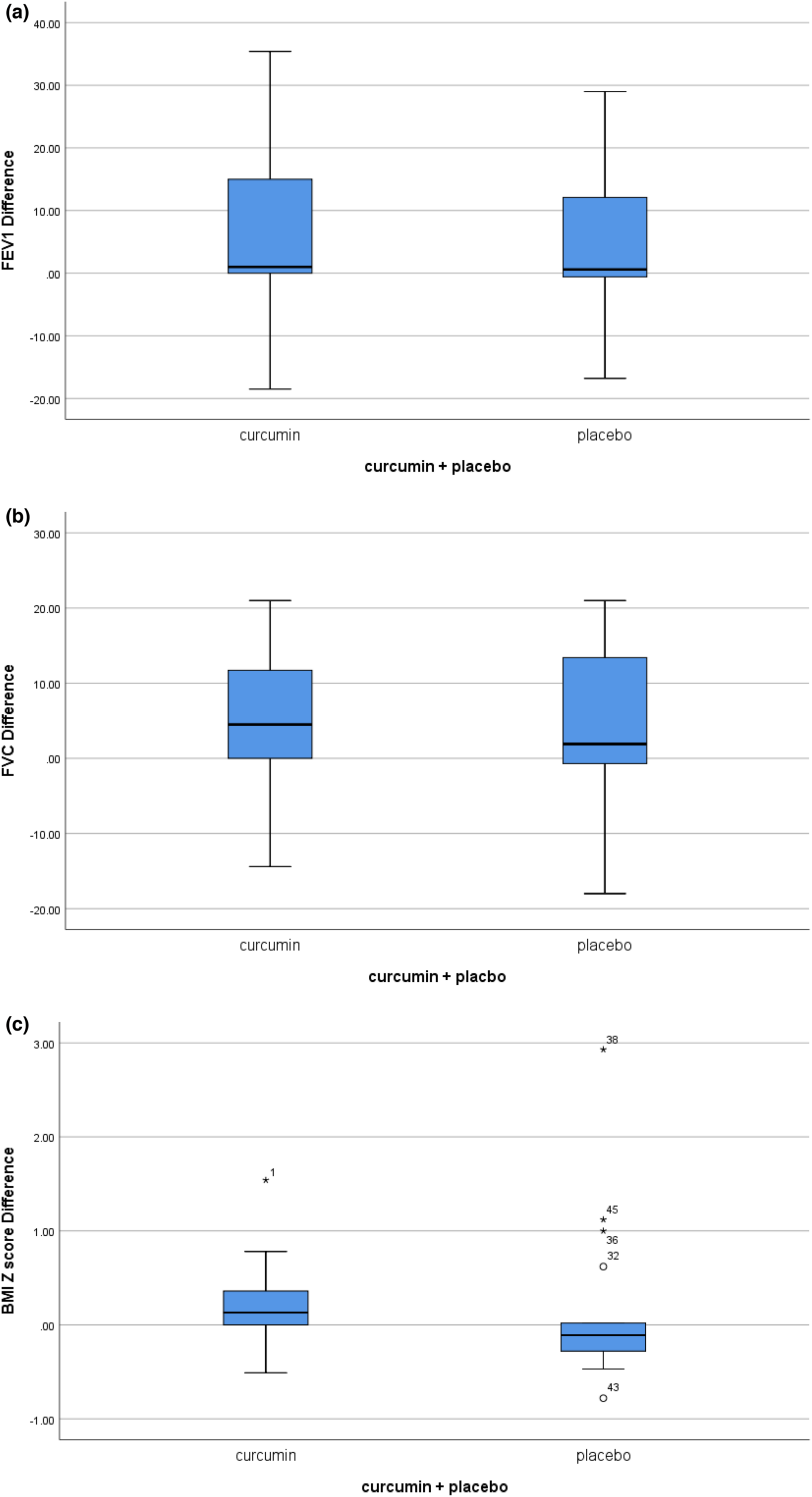
Evaluation of clinical outcome between two groups before and after the intervention. Adjusted mean changes of FEV1 percent in the curcumin and placebo groups did not show any significant changes (a), adjusted mean changes of forced vital capacity percent in the curcumin and placebo groups did not show any significant changes (b), adjusted mean changes of body mass index (BMI) *z*‐score in curcumin and placebo groups showed that BMI *z*‐score improved significantly in the curcumin group from baseline by independent *t*‐test, but intra‐group changes comparison was not significant (c).

The adjusted mean change (SE) in FEV1 increased by 5.96 ± 2.41% in the curcumin group and by 4.28 ± 3.03% in the placebo group. Although the percent increase was significant in the curcumin group (*p* = .02), the differential increase between the two groups was not different (Figure 3a). The adjusted mean change (SE) in forced vital capacity (FVC) increased by 5.24 ± 1.76 mL in the curcumin group (*p* = .006). On the other hand, in the control group, FVC increased by 4.40 ± 2.75 (*p* > .05) In addition, there was no difference between the two groups (Figure 3b).

The adjusted mean change (SE) in BMI *z*‐scores increased by 0.23 ± 0.17 in the curcumin group (*p* = .003) and 0.14 ± 0.18 in the placebo group (*p* > .05). Again, there was no difference between the two groups (Figure 3c; Table S2).

### 3.5 Comparison of anti‐microbial effects

Evaluation of the pharyngeal swab cultures of patients with CF showed that the mean (SD) *Pseudomonas* colony count was 64.03 ± 47.03 CFU mL^−1^ before the study in the curcumin group and 46.66 ± 20.81 CFU mL^−1^ in the placebo group. However, after the intervention, it decreased in the curcumin group (*p* = .01).

### 3.6 Comparison of quality of life

Regarding the effects of curcumin on the patient's clinical symptoms, the overall QOL, as well as the subscales of physical activity (*p* = .03), emotional function (*p* = .01), pulmonary function (*p* = .009), and body image (*p* = .02), improved from the parent's point of view. Curcumin also ameliorated the physical activity subscale from the children's point of view (*p* = .02), besides the treatment burden (*p* = .08) and role function (*p* = .04) from the adolescents and adults points of view over 14 years (Tables [Link], [Link]).

## 4 DISCUSSION

Curcumin is one of the polyphenolic compounds of turmeric. In the present study, nano‐curcumin at a maximum dose of 80 mg/m^2^ of body weight resulted in improvements in several aspects of systemic and gastrointestinal inflammation in children with CF after 3 months of follow‐up. Curcumin also led to improved pulmonary function and QOL scores.

### 4.1 Anti‐inflammatory effects of nano‐curcumin

In the current study, nano‐curcumin resulted in reduced inflammation, characterized by decreased levels of serum hs‐CRP levels and increased levels of IL‐10. hs‐CRP is a positive acute‐phase protein, which is synthesized by the liver under inflammatory conditions (Jain et al., 2011). In patients with CF, it has been suggested to be a predictor of disease severity and pulmonary exacerbation (Matouk et al., 2016). Moreover, in non‐CF bronchiectasis, a positive correlation has been observed between CRP and the high‐resolution computed tomography (HRCT) score (Hsieh et al., 2013). Although there is no similar study on patients with CF, one report showed that curcumin at a dose of 1500 mg for 8 weeks could significantly decrease the hs‐CRP levels in patients with ulcerative colitis (Sadeghi et al., 2020). Moreover, in patients undergoing angioplasty, nano‐curcumin at the same dose caused a significant decrease in hs‐CRP levels (Helli et al., 2021).Besides, the systematic review had shown that curcumin improved the severity of inflammation including asthma, inflammatory bowel disease, and juvenile idiopathic arthritis (Heidari et al., 2022). By using nano‐curcumin in COVID‐19 patients, although it did not have a significant effect on CRP, hs‐CRP, and IL‐6, it decreased the gene expression of IL‐6 (Shojaei, Foshati, et al., 2023).

Finally, in an in vitro cell model, curcumin reduced the death of cellular damage of Alveolar type II (ATII) cells and decreased the levels of pro‐inflammatory cytokines (TNF‐α, IL‐6, and CRP) in the serum of patients with COVID‐19 (Rocha & de Assis, 2020).

The most important effect of nano‐curcumin on reducing the inflammatory process was related to the suppression of nuclear factor kappa B (NF‐κB) pathway activation, which led to a decrease in the expression of inflammatory genes, including cyclooxygenase, and then reduced CF inflammation by inhibiting NF‐κB inhibitor (IκB) phosphorylation (Shakibaei et al., 2007). Besides, nano‐curcumin released IL‐10, which is an anti‐inflammatory agent reducing the inflammatory process (Mollazadeh et al., 2019).

### 4.2 Antimicrobial effects of nano‐curcumin

The current results showed that the administration of curcumin led to reduced *Pseudomonas* infections in pharyngeal swab culture specimens. The antiviral and antibacterial effects of curcumin have been investigated extensively: cellular and human studies have shown that curcumin can be effective in reducing *Pseudomonas* colonies and biofilms (Praditya et al., 2019). In a study by Kamurai et al. (2020), curcumin at a dose of 50 μg/mL could inhibit *Pseudomonas* colonies. Moreover, another study by Sharifian et al. (2020) evaluated the effect of nano‐curcumin on the formation of biofilm of this organism and found that nano‐curcumin at a concentration of 25 μg/mL inhibited biofilm formation in *P. aeruginosa*. Besides, the use of curcumin in combination with other antibiotics, including ciprofloxacin, greatly reduced the need for the required dose of antibiotics to inhibit these bacteria. These effects of curcumin were attributed to the bacterial cellular leakage of proteins and deoxyribonucleic acid (DNA) by curcumin. However, the appropriate dose of curcumin to inhibit *Pseudomonas* varies from 30 to 500 μg/mL (Dai et al., 2022).

The main reason for the mentioned effects seems to be the inhibition of bacterial DNA transcription or gene expression, reduction of bacterial motility, and destruction of the bacterial cell wall by curcumin (Adamczak et al., 2020). Besides, *Pseudomonas* biofilm adhesion and production were found to be inhibited in in vitro studies (Xue et al., 2020). Although there was no statistically significant inter‐group difference, it seems that the reduction in total colony forming units/mL of *Pseudomonas* seen in the current study was clinically important in patients with CF as *Pseudomonas* lung infection have a strong relation with mortality and morbidity (Moore & Mastoridis, 2017) and this result might be related to the small number of patients who had been infected by *Pseudomonas*.

### 4.3 Effects of nano‐curcumin on pulmonary function

In the present study, there were remarkable positive changes in the FEV1 and FVC, based on the evaluation of pulmonary function by spirometry, in patients receiving curcumin. It should be noted that FEV1 has been proposed as a major prognostic factor in the pulmonary evaluation of patients with CF. It is used as a method to assess the severity of disease and response to new treatments. Besides, it may be used as a predictor of the mortality rate of patients (Hulzebos et al., 2014). CF is a disease with a combination of obstructive and restrictive lung involvement.

In a study by Jusufovic et al. (2017), which evaluated the effect of curcumin plus inhaled corticosteroids in adults with known asthma, a significant change in FEV1 was observed in the curcumin group after 2 months. Moreover, in a study by Khdair et al. (2019) involving obese asthmatic patients with the chronic bronchial disease, curcumin improved the FEV1 and the FEV1/FVC ratio.

Spirometry is one of the methods for assessing pulmonary function. Malnutrition reduced BMI, and *Pseudomonas* infection has been suggested as other influential factors in pulmonary function (Kerem et al., 2014). The positive effects of curcumin on pulmonary function in the current study can likely be attributed to the improvement of growth indices and reduction of *Pseudomonas* infection in the curcumin group.

### 4.4 Effects of curcumin on the gastrointestinal inflammation

The present study showed that nano‐curcumin significantly reduced the level of fecal calprotectin. So far, no study has investigated the effect of nano‐curcumin on gastrointestinal inflammation in patients with CF, although several animal and human studies have been performed in other situations. The main mechanisms in these studies included the reduced messenger ribonucleic acid (mRNA) expression of inflammatory cytokines and decreased protein synthesis (Murphy et al., 2011), increased level of regulatory T cells, increased level of anti‐inflammatory IL‐10 in the ileum and mesenteric lymph nodes, increased proliferation and regeneration of intestinal cells, a change in the intestinal bacterial flora from inflammatory to anti‐inflammatory status, improved intestinal barrier (Bereswill et al., 2010; Sandoval‐Ramírez et al., 2021). In another inflammatory disease like inflammatory bowel disease curcumin have cellular targets interaction with NF‐κB, JAKs/STATs, MAPKs, TNF‐α, IL‐6, PPAR, and TRPV1 (Sardou et al., 2023).

Calprotectin is a protein, released from neutrophils and monocytes of the gastrointestinal tract during inflammation. The specificity of this parameter to detect gastrointestinal inflammation is estimated at 44%–93%, and its sensitivity is approximately 95%–100% (Rumman et al., 2014). Some studies have shown an association between the level of calprotectin and other indicators of the severity of CF, including malnutrition, pancreatic insufficiency, and pulmonary dysfunction according to spirometry, in addition to the presence of *Pseudomonas* infection (Talebi et al., 2022). It seems that nano‐curcumin can be considered a gastrointestinal anti‐inflammatory agent.

### 4.5 Effect of nano‐curcumin on quality of life

Regarding the QOL of adolescents and adults over the age of 14 years, there was a marked improvement in the subscales of the questionnaire, including respiratory symptoms, role function, treatment burden, body image, and weight gain after the intervention between the two groups. To evaluate the QOL of children with CF aged 6–13 years after the intervention, there was a significant difference in the subscale of physical activity. Considering the QOL of children from the parent's point of view based on the QOL questionnaire (completed by the parents of children aged 6–13 years), there was a significant difference with the placebo group in terms of the total score and the subscales of physical activity, emotional function, and lung function.

In a study evaluating the effects of nano‐curcumin on CF patients aged 5–18 years, 240 mg of curcumin for 6 months improved the emotional function subscale of the QOL. Besides, positive changes were seen in the physical function and educational functions of the curcumin group (Rafeey et al., 2020). In patients with migraine, 250 mg of phytosomal curcumin could cross the blood–brain barrier and decrease the neuroinflammation and neurotoxicity, and finally improve the headache and QOL in these patients (Shojaei, Sahebkar, et al., 2023). Studies have shown that a shorter duration of curcumin administration (<5 months) and the use of a curcumin type with higher bioavailability had better effects on the patient's QOL (Sadeghian et al., 2021).

The great need for brain cells for oxygen to function leads to their rapid exposure to oxidative stress. Under this condition, inflammation and neuronal degeneration occur, leading to neurological problems, such as anxiety, depression, and dementia. Curcumin, as an anti‐inflammatory agent, especially an antioxidant, increases glutathione levels and prevents secondary brain damage. It also improves the clinical symptoms by affecting the microbiota–gut–brain axis via the vagus nerve and reduces low‐grade inflammation in the gastrointestinal tract, and dysfunction in the tight junction., which can, in turn, affect the QOL and cognition (D'Cunha et al., 2019; Sadeghi et al., 2020).It seems that the improvement in QOL with a short course of nano‐curcumin treatment in this study could be attributed to the improvement of inflammatory markers and clinical signs in the curcumin group.

### 4.6 Side effect

Several clinical trials demonstrated that curcumin given at doses as high as 12 g/day over 3 months is safe, non‐toxic and tolerable (Gupta et al., 2012, 2013; Vogel & Pelletier, 1815). Besides, it can be used alone or in a combination with other drugs. In this current study, no adverse events related to participation occurred during the trial.

The limitations of this study were as follows: First, we did not perform a pharmacokinetic evaluation of nano‐curcumin, because this requires multiple blood sampling and CF patient and their family had poor adherence. Secondly, although curcumin can improve the function of CFTR protein as a corrector and potentiator, evidence of its effectiveness needs to CF be confirmed by evaluating the function of this protein isolated from the nasal epithelial cells or performing a sweat test.

The strengths of this study were as follows: First, two groups were well matched with a baseline value by using a stratified randomization method. Secondly, intention‐to‐treat analysis was used for the attribution of missing values. Finally, the results were adjusted with the baseline date, as a confounding factor.

## 5 CONCLUSION

The results arising from the current study demonstrate that nano‐curcumin could be considered to be an effective nutritional compound in patients with CF, with beneficial effects on inflammation, lung function, and QOL. Further studies are required to substantiate these findings and to evaluate the longer‐term effects of this intervention.

## FUNDING INFORMATION

The work financial support of the project was provided by Mashhad University of Medical Sciences, Mashhad, Iran. The funder was not involved in the study design, data analysis, and interpretation, or writing of the manuscript.

## CONFLICT OF INTEREST STATEMENT

The authors declare no conflict of interest.

## Supporting information


Table S1.

Table S2.

Table S3.

Table S4.

Table S5.

## Data Availability

The data supporting this study's findings are available on request from the corresponding author. The data are not publicly available due to privacy or ethical restrictions.
