# Supplementary material for: Adjunctive nano‐curcumin therapy improves inflammatory and clinical indices in children with cystic fibrosis: A randomized clinical trial
Source: Food Sci Nutr. 2023 Mar 28;11(6):3348–57. doi: 10.1002/fsn3.3323 (PMC10261803; doi:10.1002/fsn3.3323)
Supplement: Supplementary file 1 — Table S1. [file FSN3-11-3348-s005.doc]

| P value  Between  group | Changes^$^ | P value  Within group | After intervention | Before intervention | Sub group | variables | |
| --- | --- | --- | --- | --- | --- | --- | --- |
| 0.1* | -1.13(-7 - 6.51) | 0.10^&^ | 5.50(3-15.75) | 11.55(3.22-20.75) | curcumin | Interleukin 6  Pg/L | Systemic inflammation |
|  | 0.55(-1.20 – 0.49) | 0.61^&^ | 10(2.7-25) | 10(2.7-26) | placebo |  |  |
| 0.01* | 8.1(4.35 - 9.49) | 0.001^&^ | 13(9.5-16.5) | 5(1.9-11) | curcumin | Interleukin 10  Pg/L |  |
|  | -0.21(-3.18 -.2.91) | 0.91^&^ | 12(7-16) | 12(7-17) | placebo |  |  |
| 0.01* | -0.14(-0.6-0.26) | 0.13^&^ | 1.48(0.65-2.08) | 1.48(0.77-2.4) | curcumin | hs CRP  mg/L |  |
|  | 0.91(-0.06-1.34) | 0.14^&^ | 2.2(1.3-5) | 1.63(0.88-5) | placebo |  |  |
| 0.2** | 0.34±0.12 | 0.56^#^ | 3.41±0.73 | 3.34±0.72 | curcumin | Pharyngeal soap  (Neutrophil count) | Pulmonary inflammation |
|  | -0.23±0.10 | 0.08^#^ | 3.21±0.41 | 3.36±0.49 | placebo |  |  |
| 0.03* | -18.95(-75.74-17.30) | 0.001^&^ | 95(57-160.5) | 118(48-220) | curcumin | Stool calprotectin  µg/gr stool | Intestinal inflammation |
|  | -7.15(-14.21-7.66) | 0.85^&^ | 134.5(74.75-286.25) | 128(72-326.25) | placebo |  |  |

**Supplementary Table 1: Adjusted mean changes in inflammatory indices during the study in both group (curcumin &placebo)**

** Independent t test

^#^Wilcoxon rank-sum test

^$^Data were obtained from ANCOVA test with baseline values as the covariate

Paired t test^&^

Reported based on mean ± SD or median ± IQR

hs CRP; high-sensitivity C-reactive protein
