# Supplementary material for: Adjunctive nano‐curcumin therapy improves inflammatory and clinical indices in children with cystic fibrosis: A randomized clinical trial
Source: Food Sci Nutr. 2023 Mar 28;11(6):3348–57. doi: 10.1002/fsn3.3323 (PMC10261803; doi:10.1002/fsn3.3323)
Supplement: Supplementary file 2 — Table S2. [file FSN3-11-3348-s001.doc]

| P value  (Between groups) | Changes# | P value  (Within group) | After intervention | Before intervention | Subgroup | Variable | |
| --- | --- | --- | --- | --- | --- | --- | --- |
| 0.17 | 6.68±2.11 | 0.02 | 81.69±19.21 | 75.73±22.36 | curcumin | FEV1 | Pulmonary evaluation  Spirometry (Predictive percent) |
|  | 2.83±3 | 0.18 | 71.51±20.64 | 64.10±22.04 | placebo |  |  |
| 0.11 | 5.76±1.75 | 0.006 | 77.58±17.12 | 72.34±19.04 | curcumin | FVC |  |
|  | 3.36±2.51 | 0.13 | 67.58±20.21 | 60.07±20.01 | placebo |  |  |
| 0.03^**^ | -2.69(-4.45 - -0.43) | 0.34^*^ | 108.1(102.05-110.57) | 109.9(105-111.95) | curcumin | FEV1/FVC |  |
|  | -2.69(-4.45 - -0.43) | 0.44^*^ | 97.3(89-105) | 107(88.18-112.05) | placebo |  |  |
| 0.88 | 0.23±0.1 | 0.003 | -1.46±1.50 | -1.75±1.63 | curcumin | Anthropometrics  (BMI Z-Score) | |
|  | 0.14±0.13 | 0.46 | -1.53±1.50 | -1.67±1.54 | placebo |  |  |

Supplementary Table 2: Adjusted mean changes of clinical indices during the study in both group

(curcumin &placebo)

** Man-whiney test

* Wilcoxon rank-sum test

Reported based on mean ± SD or median ± IQR

^#^Data were obtained from ANCOVA test with baseline values as the covariate

FEV1; Forced expiratory volume in the first second, FVC; Forced vital capacity, BMI; Body Mass Index
