# Supplementary material for: Adjunctive nano‐curcumin therapy improves inflammatory and clinical indices in children with cystic fibrosis: A randomized clinical trial
Source: Food Sci Nutr. 2023 Mar 28;11(6):3348–57. doi: 10.1002/fsn3.3323 (PMC10261803; doi:10.1002/fsn3.3323)
Supplement: Supplementary file 3 — Table S3. [file FSN3-11-3348-s003.doc]

| P value  Between-group | Changes# | P value  Within group | After intervention | Before intervention | Subgroup | Quality of life  (Parent’s version 6-13 years) |
| --- | --- | --- | --- | --- | --- | --- |
| 0.003 | 8.69±2.29 | 0.001 | 80.39±12.35 | 71.70±14.03 | curcumin | Physical activity |
|  | -15.35±8.33 | 0.13 | 67.76±13.7 | 83.12±19.08 | placebo |  |
| 0.01 | 17.01±4.29 | 0.001 | 83.89±12.66 | 66.87±14.40 | curcumin | Emotional function |
|  | -5±7.14 | 0.53 | 74±13.41 | 79±17.81 | placebo |  |
| 0.74* | -5.83(-8.33-13.75) | 0.88^**^ | 79.16(66.66-83.75) | 80(75-86.25) | curcumin | vitality |
|  | 1.66(-16.66-5.6) | 1^**^ | 75(66.66-91.66) | 65(60-86.33) | placebo |  |
| 0.18* | 0(-3.12-12.5) | 0.35^**^ | 81.25(75-90.62) | 87.5(62.5-87.5) | curcumin | Health Perception |
|  | -12.5(-36.45- -2.5) | 0.18^**^ | 62.5(50-87.5) | 75(50-75) | placebo |  |
| 0.92* | 0(0-15.62) | 0.10^**^ | 100(75-100) | 87.5(75-100) | curcumin | Eating disorder |
|  | 25(0-45) | 0.18^**^ | 100(62.5-100) | 75(50-95) | placebo |  |
| 0.83* | 0(-25-6.25) | 0.83^**^ | 37.5(25-75) | 37.5(25-75) | curcumin | Weight |
|  | -25(-25- -5) | 0.15^**^ | 25(25-75) | 75(25-95) | placebo |  |
| 0.68 | 3.20±1.15 | 0.72 | 56.62±17.49 | 65.04±18.53 | curcumin | Treatment burden |
|  | -16.66±13.17 | 0.27 | 56.66±29.69 | 73.33±25.27 | placebo |  |
| 0.02 | 8.33±5.83 | 0.17 | 71.29±23.26 | 62.96±23.08 | curcumin | Body image |
|  | -66.66±66.66 | 0.42 | 33.33±14.43 | 50±43.30 | placebo |  |
| 0.53* | 6.25(-4.16-12.5) | 0.08^**^ | 83.33(66.66-95.83) | 68.75(50-87.5) | curcumin | Educational function |
|  | 0(-9.59-11.49) | 1^**^ | 75(41.66-87.5) | 90(46.87-95.83) | placebo |  |
| 0.009 | 5.32±1.74 | 0.007 | 85.64±11.36 | 78.96±9.07 | curcumin | Respiratory  function |
|  | -5.95±4.29 | 0.18 | 75±28.34 | 80.95±32.99 | placebo |  |
| 0.65 | 5.55±3.15 | 0.09 | 85.64±11.36 | 80.09±14.33 | curcumin | Gastrointestinal  function |
|  | -5.55±5.55 | 0.31 | 88.88±9.62 | 94.44±9.62 | placebo |  |

**Supplementary Table 3: parents ‘point of view Cystic Fibrosis Questionnaire (CFQ-R)**

*Man- Whitney

**Wilcoxon rank-sum test

#Data were obtained from ANCOVA test with baseline values as the covariate

Reported based on mean ± SD or median ± IQ
