# Supplementary material for: Adjunctive nano‐curcumin therapy improves inflammatory and clinical indices in children with cystic fibrosis: A randomized clinical trial
Source: Food Sci Nutr. 2023 Mar 28;11(6):3348–57. doi: 10.1002/fsn3.3323 (PMC10261803; doi:10.1002/fsn3.3323)
Supplement: Supplementary file 4 — Table S4. [file FSN3-11-3348-s002.doc]

| P value  Between-group | Changes# | P value  (Within group) | After intervention | Before intervention | Subgroup | Quality of life  (Adultescence and adult) |
| --- | --- | --- | --- | --- | --- | --- |
| 0.16 | 9.18±9.15 | 0.35 | 76.02±18.86 | 66.83±11.04 | curcumin | Physical activity |
|  | 8.03±3.61 | 0.06 | 79.91±11.75 | 71.87±11.75 | placebo |  |
| 0.78^*^ | 0(-8.32-0) | 0.45^**^ | 83.33(97.92-62.5) | 75(75-100) | curcumin | Emotional function |
|  | 0(-22.91-0) | 0.08^**^ | 66.67(58.33-77.5) | 75(33.33-83.33) | placebo |  |
| 0.63 | -7.14±6.34 | 0.30 | 72.86±20.98 | 80±21.21 | curcumin | vitality |
|  | 0±2.83 | 1 | 76.25±15.05 | 76.25±15.52 | placebo |  |
| 0.87 | 7.14±2.83 | 0.06 | 69.04±20.24 | 61.90±15.10 | curcumin | Health Perception |
|  | 2.08±3.04 | 0.68 | 68.74±19.28 | 66.66±18.89 | placebo |  |
| 0.68 | -5.95±9.22 | 0.54 | 80.95±22.41 | 86.90±12.59 | curcumin | Eating disorder |
|  | -3.12±6.48 | 0.64 | 66.66±23.98 | 69.78±24.77 | placebo |  |
| 0.001^*^ | -8.33(-16.66-16.67) | 0.03^**^ | 100(56.25-100) | 91.66(75-100) | curcumin | weight |
|  | -4.17(-22.99-0) | 0.05^**^ | 25(25-50) | 75(75-100) | placebo |  |
| 0.008 | -7.85±3.05 | 0.04 | 63.57±10.69 | 55.71±8.38 | curcumin | Treatment burden |
|  | -5.62±2.69 | 0.05 | 55±11.95 | 60.63±12.08 | placebo |  |
| 0.40 | 9.52±4.61 | 0.08 | 66.66±31.18 | 57.14±35.49 | curcumin | Body image |
|  | -4.16±2.22 | 0.10 | 53.12±25.17 | 57.29±26.51 | placebo |  |
| 0.86 | 5.71±4.28 | 0.23 | 75.71±18.35 | 70±10.80 | curcumin | Social function |
|  | -5.62±4.27 | 0.22 | 52.50±20.70 | 58.61±19.62 | placebo |  |
| 0.79^*^ | 7.14(0-7.14) | 0.34^**^ | 75(51.79-88.39) | 67.76(35.7-82.14) | curcumin | Respiratory symptoms |
|  | 1.78(-17.85-13.57) | 0.49^**^ | 75(72.32-79.64) | 71.43(64.29-85.71) | placebo |  |
| 0.27 | -3.57±9.42 | 0.71 | 82.14±21.20 | 85.71±14.20 | curcumin | Gastrointestinal symptoms |
|  | -3.12± 4.43 | 0.85 | 68.75±19.28 | 71.87±20.86 | placebo |  |
| 0.04 | 11.06±7.02 | 0.15 | 77.67±18.70 | 66.07±21.88 | curcumin | Role function |
|  | -4.37± 3.19 | 0.21 | 65.78±19.51 | 70.15±23.55 | placebo |  |

Supplementary 3: Adolescence and Adult ‘point of view Cystic Fibrosis Questionnaire (CFQ-R)

*Man-Whitney

**Wilcoxon rank-sum test

#Data were obtained from ANCOVA test with baseline values as the covariate

Reported based on mean ± SD or median ± IQ
