# Supplementary material for: Adjunctive nano‐curcumin therapy improves inflammatory and clinical indices in children with cystic fibrosis: A randomized clinical trial
Source: Food Sci Nutr. 2023 Mar 28;11(6):3348–57. doi: 10.1002/fsn3.3323 (PMC10261803; doi:10.1002/fsn3.3323)
Supplement: Supplementary file 5 — Table S5. [file FSN3-11-3348-s004.doc]

| P value  Between-group | Difference# | After intervention | Before intervention | P value  Within group | Subgroup | Quality of life  (children 6-13 years) |
| --- | --- | --- | --- | --- | --- | --- |
| 0.02* | 0(-4.16-13.54) | 87.5(76.66-100) | 87.5(60.41-97.91) | 0.34** | curcumin | Activity |
|  | -27.08(-36.45- -2.08) | 66.6(50-80) | 87.5(87.5-100) | 0.14** | placebo |  |
| 0.16 | 5.23±2.23 | 80.19±12.17 | 74.96±11.93 | 0.001 | curcumin | Emotional function |
|  | 9.91±2.34 | 81.25±12.23 | 78.90±21.85 | 0.82 | placebo |  |
| 0.9* | 0(0-8.33) | 91.66(70.83-100) | 83.33(62.5-100) | 0.14** | curcumin | Eating disorder |
|  | -12.5(-31.25-12.5) | 75(66.66-100) | 100(91.67-100) | 0.28** | placebo |  |
| 0.26 | 3.90±3.41 | 65.62±23.93 | 61.71±23.05 | 0.001 | curcumin | Treatment burden |
|  | 25±0.001 | 65.62±32.87 | 65.62±27.71 | 1 | placebo |  |
| 0.34* | 0(-25-18.75) | 75(56.25-100) | 75(50-100) | 1** | curcumin | Body image |
|  | 0(-37.5-0) | 50(25-100) | 100(37.5-100) | 0.31** | placebo |  |
| 0.36* | 4.66±0.36 | 65.44±17.33 | 65.07±18.07 | 0.08** | curcumin | Social function |
|  | 7.69±4.27 | 65.72±22.43 | 61.45±16 | 0.61** | placebo |  |
| 0.38* | 0(0-10.93) | 81.25(78.12-96.87) | 81.25(68.75-96.88) | 0.17** | curcumin | Respiratory symptoms |
|  | 9.37(-4.68-23.43) | 81.25(68.75-100) | 81.25(71.88-84.38) | 0.26** | placebo |  |
| 0.26* | 0(-25-18.75) | 75(62.5-100) | 75(62.5-93.75) | 0.79** | curcumin | Gastrointestinal  symptoms |
|  | 0(-4.68-0) | 100(75-100) | 81.25(75-100) | 0.31** | placebo |  |

Supplementary Table 5: children ‘point of view Cystic Fibrosis Questionnaire (CFQ-R)

*Man-Whitney

**Wilcoxon rank-sum test

#Data were obtained from ANCOVA test with baseline values as the covariate

Reported based on mean ± SD or median ± IQ
